# Histone H2AX Is Involved in FoxO3a-Mediated Transcriptional Responses to Ionizing Radiation to Maintain Genome Stability

**DOI:** 10.3390/ijms161226216

**Published:** 2015-12-16

**Authors:** Stephane Tarrade, Tanya Bhardwaj, Matthew Flegal, Lindsey Bertrand, Ilya Velegzhaninov, Alexey Moskalev, Dmitry Klokov

**Affiliations:** 1 Canadian Nuclear Laboratories, Stn 51, Chalk River, ON K0J 1P0, Canada; stephane.tarrade@etu.unilim.fr (S.T.); tanya_bhardwaj@live.com (T.B.); matthew.flegal@cnl.ca (M.F.); lindsey.bertrand@cnl.ca (L.B.); 2 Institute of Biology, Komi Science Center of RAS, 28b Kommunisticheskaya St, Syktyvkar 167982, Russia; vellio@yandex.ru (I.V.); amoskalev@list.ru (A.M.); 3 Department of Ecology, Syktyvkar State University, Syktyvkar 167001, Russia; 4 Laboratory of Genetics of Aging and Longevity, Moscow Institute of Physics and Technology, Dolgoprudny, Moscow Region 141700, Russia

**Keywords:** H2AX, FoxO3a, ionizing radiation, stress response, DNA damage signaling, genome stability

## Abstract

Histone H2AX plays a crucial role in molecular and cellular responses to DNA damage and in the maintenance of genome stability. It is downstream of ataxia telangiectasia mutated (ATM) damage signaling pathway and there is an emerging role of the transcription factor FoxO3a, a regulator of a variety of other pathways, in activating this signaling. We asked whether H2AX may feedback to FoxO3a to affect respective FoxO3a-dependent pathways. We used a genetically matched pair of mouse embryonic fibroblast *H2AX^+^*^/*+*^ and *H2AX^−^*^/*−*^ cell lines to carry out comprehensive time-course and dose-response experiments and to show that the expression of several FoxO3a-regulated genes was altered in *H2AX^−^*^/*−*^ compared to *H2AX^+^*^/*+*^ cells at both basal and irradiated conditions. *Hspa1b* and *Gadd45a* were down-regulated four- to five-fold and *Ddit3*, *Cdkn1a* and *Sod2* were up-regulated 2–3-fold in *H2AX^−^*^/*−*^ cells. Using the luciferase reporter assay, we directly demonstrated that transcriptional activity of FoxoO3a was reduced in *H2AX^−^*^/*−*^ cells. FoxO3a localization within the nuclear phospho-ATM (Ser1981) foci in irradiated cells was affected by the H2AX status, as well as its posttranslational modification (phospho-Thr32). These differences were associated with genomic instability and radiosensitivity in *H2AX^−^*^/*−*^ cells. Finally, knockdown of *H2AX* in *H2AX^+^*^/*+*^ cells resulted in FoxO3a-dependent gene expression patterns and increased radiosensitivity that partially mimicked those found in *H2AX^−^*^/*−*^ cells. Taken together, our data suggest a role for FoxO3a in the maintenance of genome integrity in response to DNA damage that is mediated by H2AX via yet unknown mechanisms.

## 1. Introduction

Genome integrity is constantly challenged by endogenous and exogenous DNA damaging stresses, such as exposures to reactive oxygen species originating from oxidative cellular metabolism, to external chemical and physical agents, to background and medical ionizing radiation. Processes such as aging, tumorigenesis, age-related diseases are regulated by a multitude of genetic factors controlling DNA integrity. This control is executed via several intricately related pathways, such as DNA damage signaling, cell cycle checkpoints, DNA repair, and apoptosis [1,2]. Ataxia telangiectasia mutated (ATM) is a key factor that senses DNA damage and phosphorylates its main downstream target histone H2AX to form γH2AX, leading to the activation of p53 and the execution of the downstream programs of cell cycle arrest, DNA repair or apoptosis [3,4,5]. The ATM-γH2AX-p53 axis is a canonical DNA damage pathway that regulates the cellular response to DNA damage and, depending on the context, determines the outcome.

Among other factors shown to play a key role in regulating cellular stress responses the most important is a transcription factor FoxO3a belonging to the Foxo transcription factors family [6,7,8]. The mammalian Foxo transcription factors have been implicated in a variety of crucial and diverse cellular processes, transcriptionally regulating apoptosis, cell cycle, DNA repair, glucose metabolism, cellular differentiation and other biological functions [9]. They were also found at chromosomal translocations in human tumors, indicating their role in tumor suppression [10]. One of the most striking features of FoxO3 is the prominent role it plays in longevity. In *C. elegans* and *D. melanogaster*, Foxo orthologs DAF-16 and dFoxo, respectively, were shown to increase lifespan by transcriptionally activating genes involved in resistance to oxidative stress, pathogens and damage to proteins and by facilitating chromatin remodeling [11,12,13]. Epidemiological studies showed that the *Foxo3a* genotype in humans is also strongly associated with longevity [14,15,16]. Recent evidence suggested that the mechanism by which FoxO3 activates the transcription of its target genes is mediated by the chromatin remodeling complex SWItch/Sucrose Non-Fermentable (SWI/SNF) that relaxes the chromatin to initiate transcription [13].

There is a link between aging/longevity and genomic instability. Both H2AX and FoxO3a play important roles in these processes. Importantly, FoxO3a has been shown, in addition to its well known transcriptional regulation of stress response genes, to directly interact with ATM to trigger all downstream canonical DNA damage signaling including phosphorylation of H2AX [17,18]. γH2AX is known to exert a positive feedback effect on maintaining and amplifying ATM activity via MDC1 [19]. Would it be sensible to assume that H2AX or its phosphorylated form may also impact FoxO3a in a similar feedback manner? This question becomes even more appropriate given the fact that the regulation of longevity in worms by chromatin modifications was dependent on Foxo [20].

Therefore, in this study we examined whether H2AX may play a role in the transcription of genes regulated by FoxO3a. Additionally, we studied the transcriptional responses of these genes to ionizing radiation in comprehensive dose-response and time-course experiments in the context of the presence or absence of histone H2AX. We show that both baseline and radiation-modulated expression of several genes is affected by the H2AX status. Results of experiments examining direct FoxO3a transcriptional activity, FoxO3a post-translational modification and intracellular FoxO3a localization all show that FoxO3a behavior is substantially changed in the *H2AX^−^*^/*−*^ compared to *H2AX^+^*^/*+*^ cells. Finally, we show that these differences were accompanied by increased genomic instability and radiosensitivity and that knockdown of *H2AX* in *H2AX^+^*^/*+*^ cells resulted in the effects similar to those observed in *H2AX^−^*^/*−*^ cells, providing a potential link between H2AX and FoxO3a in relation to the maintenance of genome integrity.

## 2. Results

### 2.1. Characterization of the Experimental Model of H2AX^+/+^ and H2AX^−/−^ Cells

**Figure 1:**
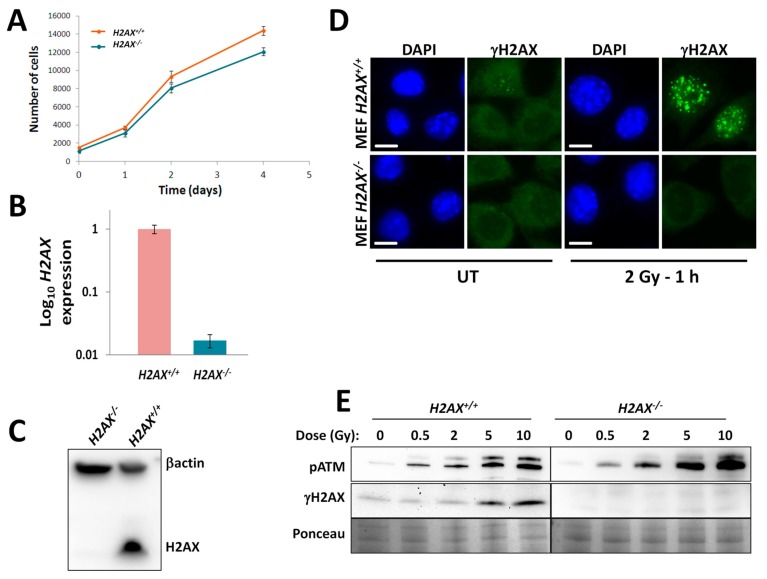
Characterization of mouse embryonic fibroblasts (MEF) *H2AX^+^*^/*+*^ and *H2AX^−^*^/*−*^ cells. (**A**) Growth curves of untreated MEF *H2AX^+^*^/*+*^ and *H2AX^−^*^/*−*^ cells; (**B**) basal *H2AX* gene expression in MEF *H2AX^+^*^/*+*^ compared to *H2AX^−^*^/*−*^ cells measured by RT-qPCR; (**C**) Western blot showing lack of H2AX protein in *H2AX^−^*^/*−*^ cells; (**D**) representative microscopic images of cells showing γH2AX foci (green fluorescence) observed at 1 h after sham-(UT, untreated) or 2 Gy irradiation of MEF cells. Nuclear perimeters were obtained by processing the DAPI (4′,6-diamidino-2-phenylindole) counterstain in Image J as described in Experimental Section and are shown as blue outlines. Data represent the mean ± SD obtained from three independent experiments; scale bar, 10 μm; (**E**) Representative Western blot images of Phospho-S1981 ATM and γH2AX obtained from whole cell protein lysates of untreated MEF *H2AX^+^*^/*+*^ and *H2AX^−^*^/*−*^ cells or 1 h after irradiation with indicated doses of γ-radiation. Note the lack of γH2AX signal in the knockout cell line. Total protein stained with Ponceau was used to control for equal loading. Data in (**A**,**B**) represent the mean ± SD obtained from three independent experiments.

We first characterized the genetically matched pair of mouse embryonic fibroblasts (MEF) *H2AX^+^*^/*+*^ and MEF *H2AX^−^*^/*−*^ cell lines in terms of (a) growth rate; (b) *H2AX* gene and protein levels; (c) ability to exert proper DNA damage response. Overall, the growth rate was slightly higher for *H2AX^+^*^/*+*^ cells; however, the difference was minimal in the first two days (Figure 1A). Cell cycle distribution was also not different between the two cell lines under control conditions and within 6 h after irradiation, followed by an accumulation of G2 cells in *H2AX^+^*^/*+*^, but not *H2AX^−^*^/*−*^ cells, indicating an aberrant cell cycle checkpoint signaling in the H2AX deficient cells (Figure S1). We confirmed that *H2AX^−^*^/*−*^ cells had negligible *H2AX* gene expression level (Figure 1B) and no H2AX protein was detected using Western blot in whole cell lysates (Figure 1C). Using immunofluorescence microscopy, we observed numerous and bright γH2AX foci in *H2AX^+^*^/*+*^ cells 1 h after 2 Gy irradiation, with only few foci were present in untreated cells (Figure 1D). No γH2AX signal was detected in *H2AX^−^*^/*−*^ cells (Figure 1D). γH2AX protein was not detected in *H2AX^−^*^/*−*^ untreated or irradiated with up to 10 Gy cells using immunoblotting, whereas in *H2AX^+^*^/*+*^ cells γH2AX protein levels were induced by irradiation in an expected dose-dependent manner (Figure 1E). Interestingly, the activation of the ATM protein by its auto-phosphorylation at Ser 1981, which is one of the earliest molecular responses to DNA damage, was not affected in *H2AX^−^*^/*−*^ MEFs (Figure 1E). Since H2AX is the main direct target for activated ATM in response to DNA damage, this observation further confirms that the lack of γH2AX induction seen is not due to an inability to phosphorylate histone H2AX, but rather to the lack of H2AX. Altogether, this data validated the usefulness of this cell model to examine a potential role of H2AX in FoxO3a-regulated cellular stress responses.

### 2.2. Transcriptional Responses of FoxO3a-Regulated Genes to Irradiation in H2AX^−/−^ vs. H2AX^+/+^ Cells

We selected 15 genes that are known to be transcriptionally activated by FoxO3a and that represent several cellular pathways summarized in Table 1 (3 genes failed validation as described below). We also included two genes, *Ddb2* and *Mdm2*, which have not been directly shown to be regulated by FoxO3a. However, the Ddb2 protein is a partner of Ddb1 in the nucleotide excision repair and may represent a good candidate target for FoxO3a [21], while *Mdm2* is intricately involved in the p53-pathway regulation in response to ionizing radiation [22]. We designed five pentaplex qPCR assays with 3 genes of interest and 2 housekeeping genes in each assay. The use of two, not one, housekeeping genes (*Actb* and *GAPDH*) minimizes inaccuracies in gene expression normalization. Cell cycle effect on housekeeping genes are also negligible since growth rates of the two cell lines were similar within 48 h (Figure 1A). Validation experiments comparing standard curves generated by singleplex *vs.* pentaplex assays and reactions with temperature gradients showed that some of the assays could not be reliably used within the pentaplex assays. Those genes were excluded from the subsequent work. The summary of the assay validation is given in Table S1 together with the details of primers and probes.

**Table 1:** FoxO3a-regulated genes chosen and validated for this study ^a^.

Gene	Pathway: Function and Reference
*CDKN1A*	Cell cycle: induces G1 arrest [23]
*Gadd45*α	Cell cycle: induces G2 arrest [24]
*Bcl2l11*	Apoptosis: bcl-2 pro-apoptotic family member [25,26]
*Bbc3*/*Puma*	Apoptosis: bcl-2 pro-apoptotic family member [27,28]
*Ddit3*/*Chop*/*Gadd153*	Apoptosis: [29]
*Gadd45*α	DNA repair: nucleotide excision repair [24]
*Ddb1*	DNA repair: nucleotide excision repair [30]
*Ddb2*	DNA repair: nucleotide excision repair [21]
*Mdm2*	DNA damage signaling [22]
*Sod2*	Anti-oxidant defense: detoxification of superoxide [31]
*Cat*	Anti-oxidant defense: detoxification of peroxides [32]
*Hspa1b*	Stress response: chaperone [33]

^a^ *Ddb2* and *Mdm2* are not known yet to be regulated directly by FoxO3a. However, Ddb2 is a smaller subunit of the DDB (DNA Damage Binding) complex consisting of Ddb1 and Ddb2; therefore, a correlation with *Ddb1* regulation is expected. Mdm2 is a crucial component of the p53 pathway transcriptionally activated in response to ionizing radiation.

**Figure 2:**
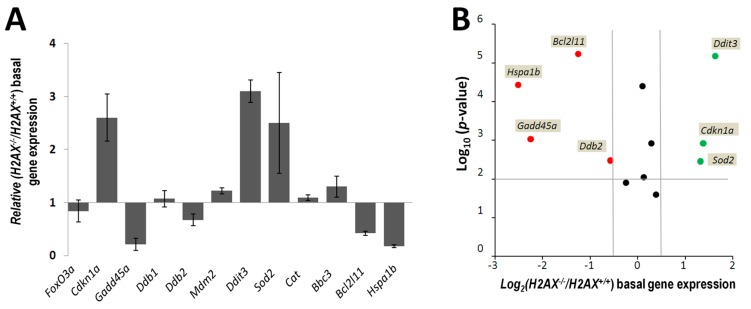
H2AX affects basal expression levels of genes regulated by FoxO3a. (**A**) Gene expression levels in *H2AX^−^*^/*−*^ relative to *H2AX^+^*^/*+*^ MEFs were measured in untreated exponentially growing cells using RT-qPCR as described in Experimental Section. Relative expression was calculated using the ∆*C*_t_ method. A value of 2^−∆∆*C*t^ > 1 indicates elevated expression of a gene, whereas a value of 2^−∆∆*C*t^ < 1 reflects decreased gene expression. Data represent the mean ± SD obtained from three independent experiments; (**B**) Volcano plot comparing altered basal gene expression in untreated *H2AX^−^*^/*−*^ relative to *H2AX^+^*^/*+*^ MEFs. The *X* axis represents the log_2_ ratio of the normalized gene signal (mean of the three experiments) of the *H2AX^−^*^/*−*^ compared to *H2AX^+^*^/*+*^ cells. The *Y*-axis is the −log_10_ *p*-value obtained from the Student’s *t* test comparing the two groups. Thresholds for gene expression regulation are set up to 1.5- and −1.5-fold. Genes that are above the *p* = 0.01 threshold were considered statistically significantly altered and their names are shown next to the data points. Red, green and black dots signify down-regulated, up-regulated and not altered genes, respectively.

Then we measured the basal levels of the selected genes in the two cell lines using RT-qPCR. We found that the expressions of *Cdkn1a*, *Ddit3* and *Sod2* were significantly higher in *H2AX^−^*^/*−*^ compared to *H2AX^+^*^/*+*^ cells (2.6-, 3.1- and 2.5-fold, respectively; Figure 2A). Four genes were down-regulated to various extents, from moderate for *Ddb2* (1.5-fold) and *Bcl2l11* (2.3-fold) to a high extent for *Gadd45*α (4.8-fold) and *Hspa1b* (5.7-fold) (Figure 2A). The volcano plot in Figure 2B shows that these alterations, with a threshold set at 1.5-fold, for all 7 genes (out of 12 analyzed) were statistically significant at *p* < 0.01 using Student’s *t*-test. Interestingly, the relative *H2AX^−^*^/*−*^ *vs.* *H2AX^+^*^/*+*^ expression of *Foxo3a* itself in untreated cells was not altered (Figure 2A).

**Figure 3:**
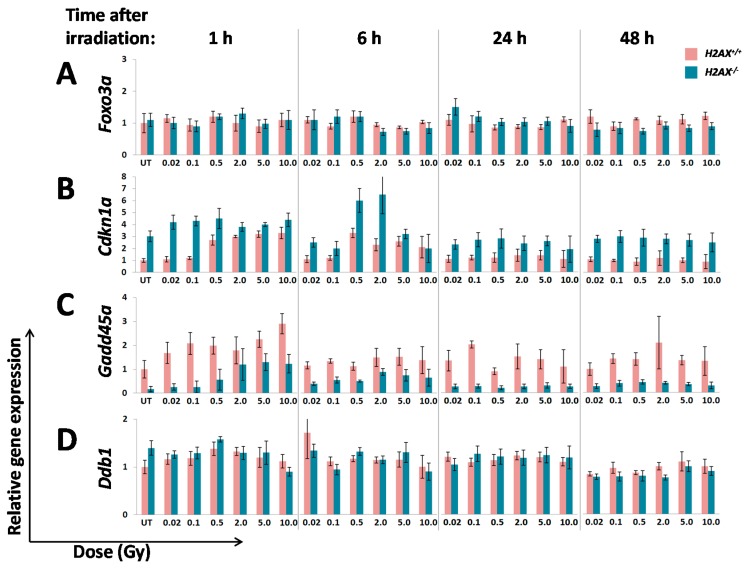

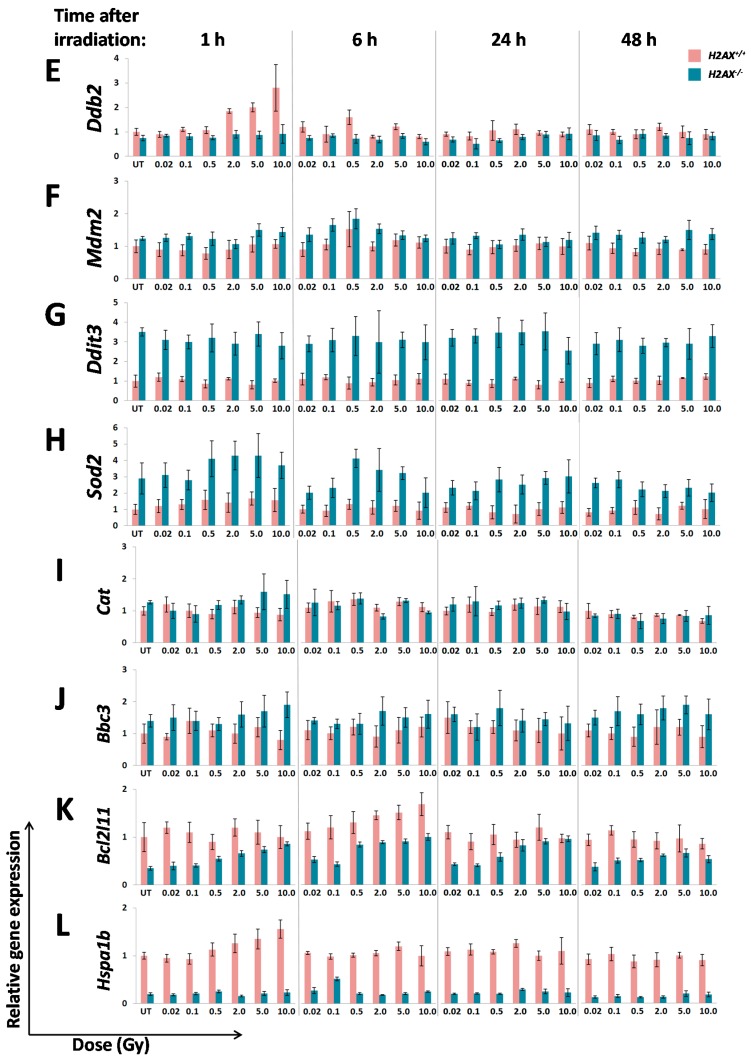
Radiation dose responses and time courses of the expression of genes regulated by FoxO3a in *H2AX^+^*^/*+*^ and *H2AX^−^*^/*−*^ MEFs. Cells were irradiated with the indicated doses of γ-radiation and at 1, 6, 24 and 48 h RNA was extracted and the expression of *Foxo3a* (**A**); *Cdkn1a* (**B**); *Gadd45*α (**C**); *Ddb1* (**D**); *Ddb2* (**E**); *Mdm2* (**F**); *Ddit3* (**G**); *Sod2* (**H**); *Cat* (**I**); *Bbc3* (**J**); *Bcl2l11* (**K**); and *Hspa1b* (**L**) were measured using RT-qPCR as described in Experimental Section. The expression values shown are relative to corresponding untreated controls and were calculated using the ∆*C*_t_ method as described in Figure 2. Data for each time-point had its own control, however, shown are only 1 h controls due to space limitation. Data represent the mean ± SD obtained from three independent experiments.

In our dose-response experiments we used a wide range of doses from as low as 2 mGy to 10 Gy. Since it is known that there may be significant differences in the kinetics of the expression of radiation-responsive genes, with some responding very early and others very late, we conducted dose-response experiments at various times after irradiation (1, 6, 24, 48 h). The results of these experiments, for all 12 genes examined, are shown in Figure 3 as expression values relative to the untreated *H2AX^+^*^/*+*^ control.

The expression of *Foxo3a* was not modulated by any of the irradiation conditions tested (Figure 3A). This may be reflective of the fact that the main means of its activation are various post-translational modifications that allow the protein shuttling between the cytoplasm and the nucleus [34].

One of the most visible differences between *H2AX^+^*^/*+*^ and *H2AX^−^*^/*−*^ cells was found for the early-response gene *Cdkn1a*. A dose-dependent increase of *Cdkn1a* expression was seen in wild type cells at 1 and partially at 6 h post-irradiation, whereas in the knockout cells the gene showed an aberrant response for both time-points (Figure 3B).

Similar differences were observed for the *Gadd45a* gene at 1 h post-irradiation. Significant increase (*p* < 0.05) in *H2AX^+^*^/*+*^ MEFs was detectable after as low as 0.1 Gy (2.1-fold) (Figure 3C). In contrast, *H2AX^−^*^/*−*^ cells responded by the induction only after the 2 Gy dose. Irrespective of induction pattern, overall levels of *Gadd45*α were substantially lower in the *H2AX* knockout cells. No radiation-induced alterations in *Gadd45*α expression were observed at 24 and 48 h in both cell lines (Figure 3C).

No changes were found for the *Ddb1* gene after irradiation at any time-point in either cell line (Figure 3D). Similar results were obtained for *Mdm2*, *Ddit3*, *Sod2*, *Cat and Bbc3* (Figure 3F–J, respectively). However, for *Ddit3* and *Sod2* there were significant differences in the baseline gene expression (Figure 2).

The *Ddb2* gene responded sharply to high radiation doses at 1 h in *H2AX^+^*^/*+*^ cells, with no response observed in *H2AX^−^*^/*−*^ cells (Figure 3E). This pattern in the wild type cells was gone by 6 h, with only the 0.5 Gy data point showing a somewhat increased *Ddb2* expression level, which however may be an artifact since it does not fit a pattern. This gene baseline expression was only slightly reduced in *H2AX^−^*^/*−*^ compared to *H2AX^+^*^/*+*^ cells (Figure 2).

*Bcl2l11* in *H2AX^+^*^/*+*^ cells demonstrated slight up-regulation with dose at 6 h post-irradiation only (Figure 3K). In contrast, the gene was upregulated by doses >0.5 Gy at all time-points in the knockout cells. However, the relative *Bcl2l11* expression level was lower in *H2AX^−^*^/*−*^ compared to *H2AX^+^*^/*+*^ cells for all the doses and time-points examined (Figure 3K). This results show that the regulation of the *Bcl2l11* gene in response to ionizing radiation is altered in *H2AX^−^*^/*−*^ compared to *H2AX^+^*^/*+*^ MEFs, with the looser control of its induction over time apparent in cells lacking the H2AX protein.

Finally, at 1 h after irradiation, the *Hspa1b* gene responded with subtle induction at high doses in the wild type cells, with *p* < 0.05 for 5 and 10 Gy only (Figure 3L). In contrast, in *H2AX^−^*^/*−*^ cells this gene did not respond to irradiation and its baseline expression was significantly lower compared to *H2AX^+^*^/*+*^ cells (Figure 2 and Figure 3L).

### 2.3. Altered FoxO3a Intracellular Localization and Post-Translational Modification in H2AX^−/−^ Cells

**Figure 4:**
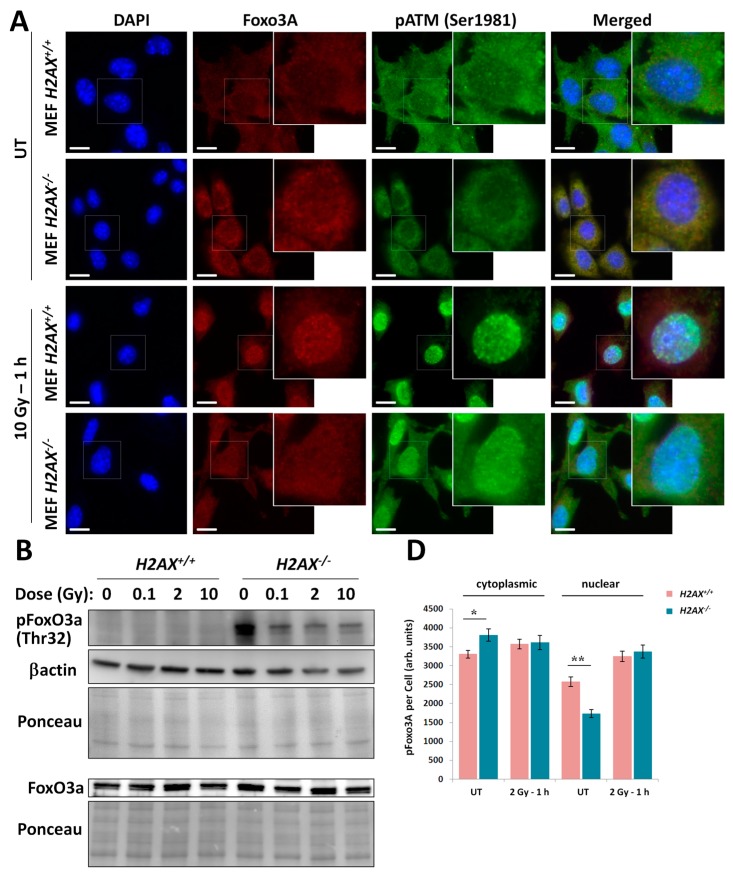

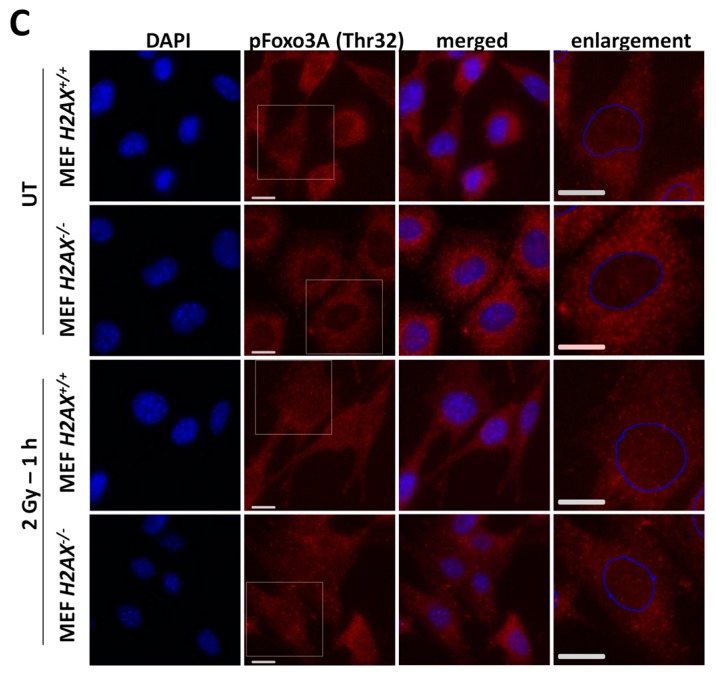
FoxO3a is differentially regulated and localized in *H2AX^+^*^/*+*^ and *H2AX^−^*^/*−*^ MEF cells. (**A**) Representative microphotographs of untreated and γ-irradiated cells showing cytoplasmic and nuclear localization of FoxO3a (red), pATM (Ser1981) (green), as well as foci formation and the two protein co-localization after irradiation in *H2AX^+^*^/*+*^ but not *H2AX^−^*^/*−*^ cells. Enlarged areas are identified by a white box and shown on the right of each channel.. Nuclei were counterstained with DAPI (blue); scale bar, 10 μm; (**B**) Representative Western blot images of pFoxO3a (Thr32) and FoxO3a obtained from whole cell protein lysates of untreated MEF *H2AX^+^*^/*+*^ and *H2AX^−^*^/*−*^ cells or 1 h after irradiation with indicated doses of γ-radiation. Ponceau and b-actin were used to control for equal loading; (**C**) Representative microphotographs of untreated *H2AX^+^*^/*+*^ and *H2AX^−^*^/*−*^ MEF cells immunostained for pFoxO3A (Thr32) (red). Nuclei were counterstained with DAPI (blue). Enlarged areas are identified by white boxes and shown on the right. Nuclear perimeters on the enlargements are shown as blue circles to allow for nuclear *vs.* cytoplasmic visualization of pFoxO3a (Thr32); scale bar, 10 μm; (**D**) Quantification of cytoplasmic and nuclear levels of pFoxO3a (Thr32) in MEF *H2AX^+^*^/*+*^ and *H2AX^−^*^/*−*^ cells expressed as arbitrary units (grey scale values) per cell. * and ** denote statistically significant difference between *H2AX^−^*^/*−*^ and *H2AX^+^*^/*+*^ cells with *p* < 0.05 and *p* < 0.01, respectively, using Student’s *t*-test (*n* = 200 cells). Data represent results from three independent experiments.

Recent data suggested that proper DNA damage response is characterized by FoxO3a colocalization with foci formed by DNA repair factors, such as phosphorylated (Ser1981) ATM (pATM), γH2AX and phospho-p53 [18]. Also, it is known that transcriptional activity of FoxO3a is regulated by its post-translational modifications [34]. Therefore, we examined intracellular localization patterns of FoxO3a, along with those of pATM under basal and irradiated conditions. Figure 4A shows representative images of these immunofluorescence microscopy experiments. We observed that in *H2AX^+^*^/*+*^ MEFs, under control conditions FoxO3a was predominantly localized to the cytoplasm. Similar pattern was found for the *H2AX^−^*^/*−*^ cells, yet cytoplasmic FoxO3a signal appeared to be higher in the knockout cells. In both cell lines, pATM nuclear signal was very low. In response to 10 Gy in wild type cells, FoxO3a re-localized into the nuclei. In contrast, nuclear re-localization was not as evident in the knockout cells. Furthermore, FoxO3a was found to form foci that co-localized with pATM foci in *H2AX^+^*^/*+*^ cells. In *H2AX^−^*^/*−*^ cells, pATM did not form foci, nor did FoxO3a, indicating abrogated DNA damage response. Interestingly, overall level of pATM did not vary substantially between the two cell lines (Figure 1E). We further examined FoxO3a phosphorylated at Thr32, a post-translational modification that is known to regulate nuclear to cytoplasmic ratio of FoxO3a. We found a substantially higher level of pFoxO3a (Thr32) in *H2AX^+^*^/*+*^ compared *H2AX^−^*^/*−*^ cells using Western blot (Figure 4B). Irradiation did not change pFoxO3a in wild type cells, whereas in *H2AX^−^*^/*−*^ cells it caused a reduction of pFoxO3a (Figure 4B). This is supported by observations of total FoxO3a levels in Figure 4A. Interestingly, levels of non-modified form of FoxO3a did not differ between the two cell lines, nor they changed after irradiation (Figure 4B), supporting similar observation for the *FoxO3a* gene expression (Figure 3A). Additionally, using immunofluorescence microscopy we directly showed that pFoxO3a was kept preferentially in the cytoplasm of *H2AX^−^*^/*−*^ cells (Figure 4C,D).

### 2.4. Increased Genomic Instability and Radiosensitivity in H2AX^−/−^ Cells 

**Figure 5:**
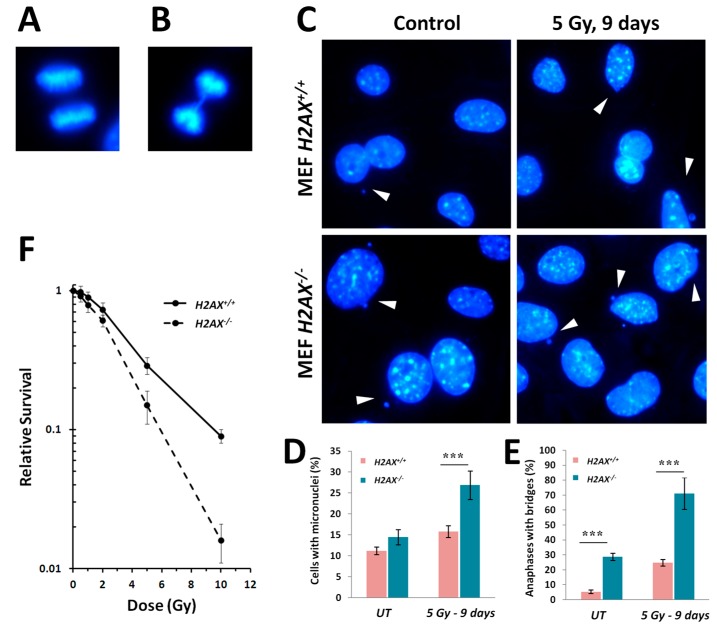
Increased genomic instability and radiosensitivity in MEF *H2AX^−^*^/*−*^ cells. Microscopic images taken using a 100× objective of a representative normal anaphase (**A**) and an anaphase bridge (**B**), the latter being an event associated with genomic instability. Cells were grown on coverslips, fixed in paraformaldehyde and stained with DAPI as described in Experimental Section; (**C**) Representative microphotographs (100× objective) of MEF *H2AX^+^*^/*+*^ and *H2AX^−^*^/*−*^ cells either sham- or 5 Gy γ-irradiated and maintained for 9 days after irradiation showing incidence of micronuclei (white arrows). Cells were fixed on coverslips and processed for DAPI staining as above; (**D**) Bar graph showing quantification of the rate of anaphase bridges in *H2AX^−^*^/*−*^ compared to *H2AX^+^*^/*+*^ untreated or 5 Gy irradiated MEFs. Cells were processed as above and anaphase bridges were scored (*n* = 200 anaphases per experiment); (**E**) Quantification of the rates of micronucleated *H2AX^−^*^/*−*^ compared to *H2AX^+^*^/*+*^ untreated or 5 Gy irradiated cells. Total of 1000 cells were scored per experiment. *** denotes statistically significant difference between *H2AX^−^*^/*−*^ and *H2AX^+^*^/*+*^ cells with *p* < 0.001 (Student’s *t*-test); (**F**) Clonogenic survival curves of *H2AX^+^*^/*+*^ and *H2AX^−^*^/*−*^ MEFs irradiated with 0, 0.5, 1, 2, 5 and 10 Gy and allowed to form surviving colonies as described in the Experimental Section. Data are plotted as a fraction of surviving cells relative to untreated control of the same genotype. Data represent the mean ± SD obtained from three independent experiments.

We evaluated genomic instability by scoring anaphase bridges and micronucleated cells (Figure 5A–C). Since genomic instability is typically considered as a delayed consequence of a DNA damaging exposure, we measured the endpoints in surviving cells 9 days following the irradiation with 5 Gy. This also minimizes the influence of conflating early responses that may not be related to genomic instability, such as apoptosis and necrosis. We found that baseline rate of anaphase bridges in *H2AX^−^*^/*−*^ cells was 5-fold higher than that in *H2AX^+^*^/*+*^ cells, whereas the baseline rate of micronucleated cells was only 1.3-fold higher in the *H2AX* knockout cells (Figure 5D,E). Nine days following 5 Gy irradiation, 71% of anaphases found had bridges in *H2AX^−^*^/*−*^ cells, with only 25% in *H2AX^+^*^/*+*^ MEFs, representing 2.8-fold difference with *p* < 0.01 (Figure 5D). We also noted that substantially fewer anaphases were present in the *H2AX^−^*^/*−*^ compared to *H2AX^+^*^/*+*^ cell population, indicating cell growth arrest or cell death effects. The rate of micronucleated cells in the irradiated cell population was also higher in the knockout compared to wild type MEFs (27% *vs.* 16%; *p* < 0.01) (Figure 5E). Finally, *H2AX^−^*^/*−*^ cells were significantly more sensitive to radiation compared to *H2AX^+^*^/*+*^ cell as assessed by their clonogenic ability (Figure 5F).

### 2.5. Effect of H2AX Knockout on FoxO3a Transcriptional Activity and Effect of H2AX Knockdown on Gene Expression and Radiosensitivity

**Figure 6:**
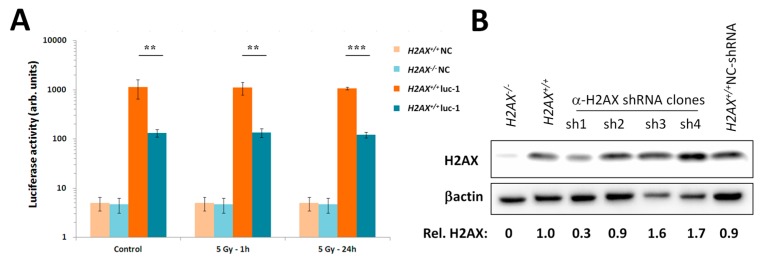

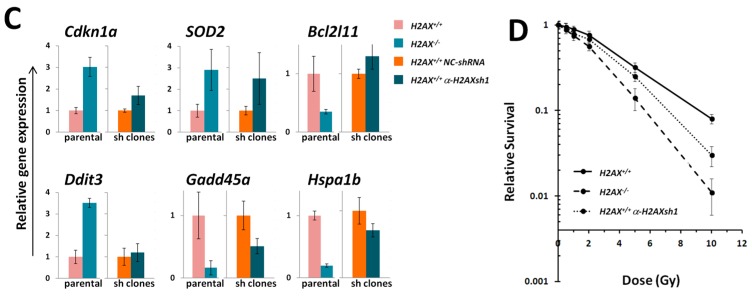
FoxO3a transcriptional activity reporter assay and H2AX knockdown experiments support involvement of H2AX in FoxO3a-mediated gene expression and radiosensitivity. (**A**) Luciferase activity in *H2AX^+^*^/*+*^ and *H2AX^−^*^/*−*^ cells stably expressing luciferase reporter gene under the control of FoxO3a-responsive promoter. The *H2AX^+^*^/*+*^ luc-1 clone and the *H2AX^−^*^/*−*^ luc-1 clone, alone with negative controls (NC), were exposed to 5 Gy γ-radiation or left untreated. Cells were lysed 1 h after irradiation and luciferase activity was measured. ** and *** denote statistically significant difference with *p* < 0.01 and 0.001, respectively (Student’s *t*-test) from three experiments; (**B**) Western blot image showing H2AX protein levels in parental *H2AX^+^*^/*+*^ and *H2AX^−^*^/*−*^ cells and in four clones of *H2AX^+^*^/*+*^ cells stably expressing shRNA targeting H2AX, along with a negative control (NC); (**C**) Effect of H2AX knockdown on the basal expression of six genes found to be differentially expressed in *H2AX^+^*^/*+*^ and *H2AX^−^*^/*−*^ cells (see Figure 3). NC denotes cells expressing non-targeting shRNA (sh); (**D**) Effect of H2AX knockdown on the clonogenic survival of MEF cell lines. Data represent the mean ± SD obtained from two independent experiments (three technical replicates in each experiment).

We directly measured transcriptional activity of FoxO3a using luciferase reporter gene assay. Using lentiviral particles, we generated cell lines that stably express a genetic cassette consisting of a FoxO3a-responsive promoter that controls activity of the luciferase gene on both *H2AX^+^*^/*+*^ and *H2AX^−^*^/*−*^ backgrounds. Figure 6A shows results of the luciferase activity measurement in *H2AX^+^*^/*+*^ and *H2AX^−^*^/*−*^ clones. We observed a significant difference in the FoxO3a transcriptional activity depending on the H2AX status, with it being substantially higher in *H2AX^+^*^/*+*^ cells (Figure 6A). We further validated our findings by generating H2AX knockdown cell lines using shRNA targeting H2AX (Figure 6B). We then used a cell line *H2AX^+^*^/*+*^ α-H2AX sh1 that had only 30% of H2AX protein found in the parental wild type cells to measure the expression of 6 genes that were found to be differentially expressed in *H2AX^+^*^/*+*^ *vs.* *H2AX^−^*^/*−*^ cells. A trend towards the pattern observed between parental wild type and knockout cells was observed in shRNA (sh) clones (negative control *vs.* sh1) for *Cdkn1a*, *SOD2*, *Gadd45a* and partially for *Hspa1b* (Figure 6C). However, *Bcl2l11* and *Ddit3* were not different between the control and sh1 shRNA clones (Figure 6C). The changes observed in the sh1 clone were sufficient to increase radiosensitivity as measured by the clonogenic survival assay (Figure 6D).

## 3. Discussion

We addressed the question of whether H2AX has a role in the FoxO3a-regulated transcriptional activities by using a cell culture model consisting of two mouse embryonic fibroblast cell lines, one of which was wild type (*H2AX^+^*^/*+*^ cells) and another was *H2AX* knockout cell line (*H2AX^−^*^/*−*^ cells). The use of these genetically matched cell lines allows the attribution of differences in measured endpoints to H2AX status. The validation of the chosen model showed not only that the H2AX transcript was not expressed and H2AX/γH2AX was not detected under control and irradiated conditions in the knockout cells, but also that the upstream event in the DNA damage response, phosphorylation of ATM at Ser 1981 (pATM), was unaffected by the *H2AX* status (Figure 1). Since FoxO3a plays an important role in ATM activation and downstream DNA damage signaling [17], this observation indicates that H2AX has no role in this step of the molecular DNA damage response. However, pATM foci did not form in *H2AX^−^*^/*−*^ cells, indicating an aberrant DNA damage response (Figure 4A). We did observe slightly lower growth rate in H2AX deficient cells. However, in the context of current work when most of the responses studied were within 48 h of irradiation, it is reasonable to assume that cell cycle differences exert minimal effect.

The fact that we found significant differences between *H2AX^+^*^/*+*^ and *H2AX^−^*^/*−*^ cells in the basal level of the expression of several genes that are targets for FoxO3a is an indication that unphosphorylated form of H2AX predominant in untreated cells, rather than γH2AX, mediates these differences (Figure 2). The altered genes include apoptosis mediator *Bcl2l11*, chaperone protein gene *Hspa1b*, cell cycle regulator *Cdkn1a*, and DNA damage and repair genes *Ddit3*, *Gadd45*α, *Ddb2*, and anti-oxidant defense gene *Sod2*. Although their role is essential for responses to stressed conditions, even under normal physiological conditions cells are continuously exposed to DNA damaging by-products of oxygen metabolism, reactive oxygen species. Therefore the defense mechanisms are never completely dormant and differences in the way low level damage is coped with may result in significant consequences. Indeed, the *H2AX^−^*^/*−*^ cells display strikingly higher levels of various markers of genomic instability [5], such as micronucleated cells and anaphase bridges, compared to wild type cells (Figure 5). The lack of H2AX has a negative impact on the cells’ ability to repair DNA damage [5] since γH2AX is known to recruit and sequester DNA repair factors at the broken DNA ends [35].

We measured transcriptional activity of FoxO3a directly by using a FoxO3a-driven luciferase reporter gene system and showed that it was lower in the absence of H2AX. In this context, it is important to point out that not all FoxO3a-dependent genes were down-regulated in H2AX knockout cells (down-regulated: *Gadd45*α, *Ddb2*, *Bcl2l11*, *Hspa1b* and up-regulated: *Cdkn1a*, *Ddit3*, *Sod2*). While it is unclear how this differential regulation may be executed mechanistically, it is known that FoxO3a can also act as a suppressor of transcription [36]. Apparently this complex transcription pattern may be chromatin context dependent. One intriguing question is whether it is possible that H2AX is concentrated at the regions of the genes (e.g., those found to be down-regulated in the H2AX deficient cells) so that it directly or indirectly recruits FoxO3a to the genes to initiate transcription. This preferential localization of H2AX does not seem completely unlikely, since transcription start sites were shown to be enriched with H2AX molecules [37]. This could explain the down-regulation of the expression of genes in cells lacking H2AX. Furthermore, the worms orthologs of *Foxo3*, *Daf-16*, is able to recruit chromatin remodeling factors to DNA for transcriptional activation [13], making the proposed sophisticated interaction between FoxO3a and genomic DNA even more feasible.

A number of significant differences in transcriptional responses to ionizing radiation that were observed in *H2AX^−^*^/*−*^ compared to *H2AX^+^*^/*+*^ cells can be categorized broadly into the following (Figure 3):

1. Inability to trigger response (*Ddb2*, *Hspa1b*)
2. Delayed/weaker induction (*Cdkn1a*)
3. Prolonged/stronger induction (*Gadd45*α, *Bcl2l11*)

In the first category, the *Ddb2* and *Hspa1b* genes, represent DNA repair [38] and protein repair systems [39]. Lack of the induction of these two genes in response to irradiation and their lower baseline levels, especially for *Hspa1b* (that encodes Hsp70 chaperone) that was 5.7-fold lower in H2AX deficiency compared to wild type genotype, may explain genomic instability observed in *H2AX^−^*^/*−*^ cells, both untreated and irradiated. Indeed, the deficiency in Hsp70 results in genomic instability in mouse embryonic fibroblasts [40], consistent with our findings.

Interestingly, *Ddb2*, in addition to its nucleotide excision repair role, was reported to suppress *Cdkn1a*/*p21* expression as a positive feedback in response to stress [41]. It follows then that lower baseline expression of *Ddb2* in *H2AX^−^*^/*−*^ cells may lead to the higher than in the wild type control expression of *Cdkn1a* (Figure 2). This may further cause an altered (induction at lower doses due to weaker control) and delayed transcriptional response of *Cdkn1a* in H2AX deficiency observed in this study (Figure 3B).

The two genes in the third category, *Bcl2l11* and *Gadd45*α, exert opposing functions in response to stress, with the former being pro-apoptotic, and the latter being a cell cycle regulator and DNA repair facilitator. Thus, stronger induction of both genes in *H2AX^−^*^/*−*^ compared to *H2AX^+^*^/*+*^ cells seems counter-intuitive. It appears then that the levels of *Gadd45*α gene induction by irradiation in *H2AX^−^*^/*−*^ cells may still be not sufficient for it to maintain its protective functions properly; rather lower than normal levels of *Gadd45*α may lead to genomic instability [42]. It is reasonable to assume that a different cellular context in *H2AX^−^*^/*−*^ cells may define how cells respond to these genes expression levels and changes.

With respect to the question how H2AX deficiency can change FoxO3a transcriptional activity, we demonstrated that phosphorylated form of FoxO3a (Thr32) is at much higher level in the knockout cells. This modification keeps the protein outside of the nucleus [34] and may prevent excessive apoptotic gene expression driven by FoxO3a [43]. This may be related to the survival pathway activation in *H2AX^−^*^/*−*^ cells to counteract increased level of DNA damage and genomic instability. One of the striking differences we found in *H2AX^−^*^/*−*^ *vs.* *H2AX^+^*^/*+*^ cells was that FoxO3a was not able to (a) efficiently translocate into the nucleus of irradiated cells; and (b) form foci that colocalize with pATM foci, pointing to another interesting possibility that transcriptional activity of FoxO3a is related to its interaction with DNA damage response proteins.

Given the increased genomic instability in H2AX*^−^*^/*−*^ cells, their long-term maintenance may have resulted in H2AX-independent changes affecting FoxO3a-dependent pathways. To rule out this possibility, we produced a cell line in which H2AX was knocked down (by 70%) by shRNA technology. Experiments conducted with this cell line within three-weeks of the knockdown (to eliminate selection of H2AX-independent changes) showed that transcriptional pattern of FoxO3a-dependent genes was partially resembling those observed in H2AX*^−^*^/*−*^ cells, suggesting that overall transcriptional changes described in this study are due to the link between H2AX/γH2AX and FoxO3a. Further studies are warranted to gain mechanistic knowledge into the details of how H2AX/γH2AX exerts its effects on FoxO3a-regulated pathways.

## 4. Experimental Section

### 4.1. Cell Cultures and Irradiations

Mouse embryonic fibroblasts (MEF) of C57BL background (kindly provided by Nussenzweig, NIH, Bethesda, MD, USA) were maintained by weekly sub-culturing in Dulbecco’s Modified Eagle’s Medium (DMEM) (HyClone, South Logan, UT, USA), supplemented with 10% foetal bovine serum (FBS) (Gibco, Invitrogen, Grand Island, NY, USA), at 37 °C in a 5% CO_2_—95% air atmosphere. Sub-confluent, exponentially growing MEF cells were used in experiments. One day prior to irradiation, 1–2 × 10^6^ cells were seeded onto 100 cm Petri dishes. The cells were irradiated at room temperature using either a γCell-200 irradiator (Atomic Energy Canada Limited, Chalk River, ON, Canada) equipped with ^60^Co γ-ray source at a dose rate of 100 mGy/min (for doses of 0.02 and 0.1 Gy) or a γCell-220 irradiator (Atomic Energy Canada Limited) equipped with ^60^Co γ-ray source at a dose rate of 4.5 Gy/min (for doses of 0.5 Gy and up). Immediately after irradiation the cell cultures were returned to the CO_2_-incubator and incubated for various times before sampling.

### 4.2. Cell Growth Assay

To determine cell growth kinetics, cells were seeded onto wells of a black CELLSTAR 96-well plate (Greiner Bio-One, Solingen, Germany) at a density of 1000 cells per well. Three wells per cell line per day of a 6-day experiment were used. Six hours later (sufficient time for cells to attach to surface), cells in the first three wells for day = 0 time-point were fixed in methanol for 60 s and stained with 0.05 μg/mL DAPI in PBS in a biological safety cabinet so that cells in the remaining wells can further be used for subsequent days. The 96-well plate was then removed back to the incubator. On subsequent days, similar procedure was performed for corresponding wells. At the end of the experiment, the plate was imaged using ImageXpress Micro high content analysis system (Molecular Devices, Sunnyvale, CA, USA) at 10× magnification. Cell numbers were determined using the “Count Nuclei” module within the MetaXpress software (Molecular Devices). Cell numbers relative to day = 0 were then determined and plotted as a growth curve.

### 4.3. Luciferase Reporter Assay

To avoid the negative influence of transduction efficiency on experimental results, stable cell lines expressing luciferase reporter construct were generated using Cignal Reporter Assay System (Qiagen, Valencia, CA, USA). To this end, 50% confluent MEF *H2AX^+^*^/*+*^ and *H2AX^−^*^/*−*^ cells were transduced with lentiviral particles containing a luciferase gene under the control of a FoxO-responsive promoter at 50 MOI for 24 h in DMEM supplemented with SureENTRY reagent (Qiagen). Twenty four hours later, DMEM with 25 μg/mL puromycin (Sigma, St. Louis, MO, USA) was added to cells and cell lines stably expressing the genetic constructs (both FoxO-luc and negative control) were generated by serial dilution and clonal expansion under puromycin selection. For irradiation experiments, cells were seeded onto wells of a 96-well plate at 5000 cells per well and irradiated a day later. Cells were then lysed in reporter lysis buffer (Promega, Madison, WI, USA) and luciferase activity was determined in triplicate whole cell lysates using the Luciferase Assay System (Promega). Luminescence was measured using Glomax 96-well plate luminescence reader (Promega) and luciferase activity was expressed as arbitrary units (equivalent of relative light units).

### 4.4. Generation of Stable H2AX Knockdown Cell Lines

Five thousand MEF *H2AX^+^*^/*+*^ cells were seeded onto wells of a 96-well plate. Next day, lentiviral particles containing a construct that encodes a shRNA hairpin targeting H2AX (Santa Cruz Biotechnology, Santa Cruz, CA, USA, sc-62464-V) were added to 50% confluent cells at 40 MOI (multiplicity of infection), along with the Polybrene supplemented DMEM. Cells were transduced for 24 h, after which puromycin was added at a concentration of 25 μg/mL. In parallel, cells were transduced with negative control lentiviral particles. Stable clones were generated by serial dilution and expansion in puromycin supplemented DMEM. Clones were screened for knockdown efficiency by Western blot.

### 4.5. RNA Extraction and cDNA Synthesis

RNA was extracted using Aurum Total RNA Mini kit (Bio-Rad, Hercules, CA, USA) as per manufacturer’s recommendations. Extracted RNA was immediately quantified and its quality evaluated using a lab-on-chip capillary automated electrophoresis system Experion (Bio-Rad). Samples with a RQI (RNA Quality Index) of 8 and above were used for further experimentation. To control for possible inhibition of reverse transcription reactions, Solaris RNA Spike Control kit was used (Thermo Scientific, Rockford, IL, USA). Briefly, a synthetic RNA molecule (Solaris™ RNA Spike Control, Rockford, IL, USA) was added to RNA samples and water control, followed by a reverse transcription reaction using iScript Reverse Transcription Supermix (Bio-Rad) as per manufacturer’s instructions. Only fresh unfrozen RNA was used. The Spike cDNA was then quantified using Solaris qPCR RNA Spike Assay (Thermo Scientific) according to the manufacturer’s instructions. The level of reverse transcription inhibition is estimated based on a comparison of C_q_ values between water and RNA samples using a formula ∆*C*_q_ = C_q(RNA sample)_ − *C*_q(water sample)_. Samples with no significant inhibition of reverse transcription (∆*C*_q_ < 3) were used for subsequent qPCR to quantify the expression levels of the genes of interest.

### 4.6. Quantitative Real-Time PCR

Pentaplex qPCR assays were designed using the online RealTimeDesign tool (www.qPCRdesign.com, BioSearch Technologies, Novato, CA, USA). Three genes of interest and two housekeeping genes were included into each qPCR pentaplex assay. The software conducts a sophisticated analysis of standard primer features and interactions between primers and probes. Dual labeled probes quenched with the Black Hole Quencher™ dye (BHQ, BioSearch Technologies, Novato, CA, USA) were used. Table S1 shows target genes included into each pentaplex assay, primer and probe sequences, melting temperatures and validation results. The pentaplex assays were experimentally validated by generating standard dilution curves for individual assays that were part of the pentaplex assays and by comparing these curves with the ones generated with the pentaplex assays. Temperature gradient reactions were also run to determine the optimum reaction conditions. All amplification reactions were conducted on a CFX96 PCR Detection System (Bio-Rad) equipped with a 5-channel optical module. The reaction mix contained 10–30 ng cDNA, derived from total RNA, primers (400 nM each), probe (200 nM), and ImmoMix master mix (Bioline Inc., Taunton, MA, USA) in a final volume of 20 μL. The following PCR cycling conditions were used: 95 °C for 10 min, 40 cycles of 95 °C for 10 s, 60 °C (or 55–65 °C gradient) for 10 s, 72 °C for 25 s. As a result of this experimental validation, three out of five pentaplex assays were reduced to quadruplex assays since *Pparg*, *Tnfsf10* and *G6pc* genes could not be reliably detected within their corresponding pentaplex assays (Table S1). Each reaction was run in triplicate and mean *C*_q_ values were used for the evaluation of the gene expression levels by comparing the results produced for the irradiated groups with those produced for the untreated control. Relative expression was calculated using ∆*C*_t_ method. Multiplexed qPCR data were normalized to the internal standards *Actin*β and *GAPDH*. Data were analyzed using CFX Manager software (Bio-Rad) and Excel (Microsoft Corp., Redmond, WA, USA).

### 4.7. Immunoblotting

MEFs were collected by trypsinization and subsequent centrifugation followed by the lysis in RIPA buffer (20 mM Tris pH 7.5, 150 mM NaCl, 1 mM EDTA, 1% NP-40, 0.5% sodium deoxycholate, 0.5% Sodium Dodecyl Sulphate containing protease and phosphatase inhibitors (Roche Diagnostics, Mannheim, Germany)). Total protein concentrations in the lysates were determined using the RC DC (reducing agent and detergent compatible) protein assay kit (Bio-Rad) by measuring absorption at 750 nm on the iMark microplate reader (Bio-Rad). For each sample, a total of 30 μg of protein was resolved on 4%–15% gradient mini-Protean precast polyacrylamide gels and transferred to PVDF membranes (Bio-Rad). After blocking with 5% skim milk in TBST (0.2% Tween-20 in TBS) for 1 h, the membranes were incubated overnight at 4 °C with the primary antibodies diluted in TBST with 5% skim milk. The primary antibodies used were monoclonal anti-γH2AX (phospho S139, Abcam, 9F3, 1:500 dilution), monoclonal anti-ATM (phospho S1981, Abcam, 10H11.E12, 1:250 dilution), polyclonal rabbit anti-H2AX (Abcam, ab11175, 1:2000 dilution), polyclonal rabbit anti-FoxO3a (Millipore; #07-702, 1:500), polyclonal rabbit anti-FoxO3a (phospho-Thr32, Cell Signaling; #9464, 1:1000), monoclonal mouse anti-b-actin (Santa Cruz Biotechnology; sc-47778, 1:3000 dilution). The membranes were then rinsed three times in TBST and incubated for 1 h at room temperature with corresponding secondary goat ant-mouse HRP-conjugated antibody (Sigma-Aldrich, St. Louis, MO, USA). Following three rinses in TBS, chemiluminescence was initiated by incubating the membranes with Immun-Star Western C reagent (Bio-Rad). Chemiluminescence reactions on membranes were imaged using Chemidoc XRS imager (Bio-Rad), followed by processing of the chemiluminescence images using ImageLab (Bio-Rad) or Image J (NIH) software applications.

### 4.8. Immunofluorescence Microscopy

Exponentially growing MEF cells were seeded onto 30 mm diameter Petri dishes containing 22 mm × 22 mm sterile glass coverslips and allowed to attach overnight. The next day, cells were irradiated using the γCell-220 irradiator as described above. One hour after the irradiation the cells were fixed in 2% paraformaldehyde/TBS for 30 min at room temperature. At the time of fixation, cell cultures were 40%–60% confluent. Cells were then rinsed in TBS and −20 °C methanol was added to cells for 1 min, followed by blocking step using TTN buffer (1% BSA, 0.2% Tween-20 in TBS) for 30 min at room temperature. For γH2AX detection, coverslips with cells were incubated with anti-γH2AX mouse antibody (phospho S139, Abcam, 2F3) diluted 1:600 in TTN for 2 h at room temperature. After rinsing with TBS and blocking with TTN for 20 min, cells were stained for 1 h at room temperature with secondary goat anti-mouse Alexa-488 antibody (Invitrogen, Life Technologies, Camarillo, CA, USA) at a 1:600 dilution in TTN. For FoxO3a and pATM detection, cells were incubated with a mixture of rabbit polyclonal anti-FoxO3a antibody (Santa Cruz Biotechnology, sc-11351) and mouse monoclonal anti-pATM antibody (phospho-Ser1981, Millipore, Temecula, CA, USA, 10H11.E12) diluted 1:100 and 1:400 in TTN, respectively. Following 2 h incubation with the primary antibodies, a mixture of goat anti-mouse Alexa-488 and goat anti-rabbit Alexa-594 antibodies (Invitrogen, Life Technologies) both diluted 1:400 in TTN was added to cells for 1 h at room temperature. For pFoxO3a detection, coverslips were incubated with anti-pFoxO3a antibody (phospho-Thr32, Cell Signaling; #9464) diluted 1:100 in TTN for 2 h, followed by a goat anti-rabbit Alexa-594 antibody (Invitrogen, Life Technologies) incubation for 1 h at room temperature. After staining with secondary antibodies, coverslips were rinsed 3 × 10 min, immersed in 0.05 μg/mL DAPI in TBS, mounted on microscope slides using Vectashield mounting medium (Vector Laboratories, Burlingame, CA, USA) and then sealed with nail polish. Slides were analyzed using a Zeiss AxioObserver Z1 fluorescence inverted microscope equipped with AxioCam iCc1 CCD camera (Zeiss, Chester, VA, USA). Five optical Z-sections per nucleus were captured using a Plan-Apochromat 63× objective and AxioVision 4.8.1 software package (Zeiss, Chester, VA, USA), and the images were generated by projection of a maximum intensity algorithm using Image J software. Images of DAPI fluorescence were used to generate the nuclear perimeter outlines by using the threshold tool in Image J. Quantification of cytoplasmic and nuclear localization of pFoxO3a (Thr32), as well as generation of merged images for FoxO3a and pATM colocalization study, was done using Image J. Two hundred cells per group per experiment were used for nuclear:cytoplasmic pFoxO3a quantification. Cells stained with secondary antibody alone were used to control green and red fluorescence signal specificity.

### 4.9. Evaluation of Genomic Instability

Exponentially growing cells in 100 mm Petri dishes were sham- or γ-irradiated with 5 Gy using the γCell-220 irradiator as described above. Cells were then maintained for 9 days with sub-culturing on days 3 and 7 post-irradiation. Media was changed every two days to avoid cytotoxicity caused by dying cells in irradiated cultures. On day 7, cells were seeded onto 22 mm × 22 mm sterile glass coverslips inside 30 mm Petri dishes and incubated for another two days reaching 40%–60% confluency (number of irradiated cells for plating had to be significantly increased compared to untreated cells due to cell cycle arrest and cell death induced by irradiation). Cells were then fixed in 2% paraformaldehyde/TBS for 30 min at room temperature, rinsed in TBS and −20 °C methanol was added to cells for 1 min. DAPI (0.05 μg/mL in TBS) was added to cells for 5 min at room temperature, followed by a rinse in TBS and mounting on microscope slides using Vectashield mounting medium (Vector Laboratories). Coverslips were sealed with nail polish and analyzed on a Zeiss fluorescence AxioObserver Z1 inverted microscope (Zeiss). Anaphase bridges and micronucleated cells were scored manually in a total of 200 anaphases and 1000 cells, respectively. Representative images of DAPI stained cells were taken using a Plan-Apochromat 63X objective, QImaging Exi Aqua Cooled CCD color camera (Qiaimaging, Surrey, BC, Canada) and Northern Eclipse V8 software package (Empix Imaging, Mississauga, ON, Canada).

### 4.10. Clonogenic Survival Assay

Exponentially growing MEF cells in 100 mm Petri dishes were detached by treatment with Trypsin-EDTA, counted and re-suspended in DMEM at a concentration of 5000 cells/mL. Several aliquots were made in 15 mL conical tubes that were then irradiated with various doses in the γCell-220 irradiator as described above. Control cells were sham-irradiated. Cells were immediately seeded onto 100 mm diameter Petri dishes at densities to yield approximately 100 colonies per plate (determined empirically in preliminary experiments). After 10 days of incubation under normal conditions, plates were fixed and stained in 50% methanol with crystal violet. Plates were scored manually using Colony Counter SC6 (Bibby Scientific Ltd., Stone, UK) and colonies that contained >100 cells were counted as survivors. The plating efficiency in the control was ~60%, and the numbers of colonies in experimental groups were normalized to the control. Each experiment was performed in triplicate, and the means ± SD of the surviving fraction of cells were determined.

### 4.11. Statistical Analysis

Experiments were repeated at least three times, with the exception of the clonogenic survival experiment with anti-H2AX-shRNA-sh1 cell line for which the experiment was repeated two times with 3 technical replicates per experiment, and values plotted are means of the biological replicates. Standard deviations were used to evaluate errors. Treatment groups were compared using two-tailed Student’s *t*-test. A significance threshold was set up to *p* < 0.05.

## 5. Conclusions

Overall, we observed a complex differential pattern of gene responses to ionizing radiation in *H2AX^−^*^/*−*^ compared to *H2AX^+^*^/*+*^ cells. This was accompanied by differential intracellular localization of FoxO3a and its post-translational modification depending on the H2AX status. Since these transcriptional responses are regulated mostly by the FoxO3a transcription factor in normal cells, our results point to the role of H2AX in the FoxO3a-regulated transcriptional responses. This suggestion is supported by direct measurements of FoxO3a transcriptional activity using the luciferase reporter gene system and by observing similar responses upon down-regulation of H2AX by shRNA in wild type cells. The mechanisms of how the status of H2AX exerts its effect on FoxO3a dependent pathways are not clear and our results do not provide a direct link between H2AX/γH2AX and FoxO3a. However, based on the complex responses observed where some genes are up-regulated, whereas others are down-regulated, with altered dose-responses and the kinetics of the responses, it is sensible to assume that the mechanisms are at least as complex. It appears that each pathway may have its own mechanism for H2AX to exert its effect on the way FoxO3a regulates the response. Furthermore, these differences are likely to be associated with genetic instability and increased radiosensitivity in H2AX deficient cells.

## Supplementary Materials

Supplementary materials can be found at http://www.mdpi.com/1422-0067/16/12/26216/s1.
