# Supplementary material for: Histone H2AX Is Involved in FoxO3a-Mediated Transcriptional Responses to Ionizing Radiation to Maintain Genome Stability
Source: Int J Mol Sci. 2015 Dec 16;16(12):29996–30014. doi: 10.3390/ijms161226216 (PMC4691159; doi:10.3390/ijms161226216)
Supplement: Supplementary file 1 [file ijms-16-26216-s001.pdf]

Supplementary Material: Histone H2AX Is Involved in FoxO3a-Mediated Transcriptional Responses to Ionizing Radiation to Maintain Genome Stability

Stephane Tarrade, Tanya Bhardwaj, Matthew Flegal, Lindsey Bertrand, Ilya Velegzhaninov, Alexey Moskalev and Dmitry Klokov

Table S1. Details of five pentaplex qPCR assays used in this study.

5plex ID	Gene Name/ID [a]	Sequence/Probe Type	T_m (°C)	Validation Result
#1	*CDKN1A*/ NM_007669	For: 5′-ACCGTGGGTGTCAAAGCA-3′	67	Passed
		Rev: 5′-GTGCGGGCTAAGGGTAGA-3′	65	
		Probe: FAM—BHQ1	-	
		5′-TTAGTGGGTCTGACTCCAGCCC-3′	69	
	SOD2/ NM_013671	For: 5′-CGCCTGCCATGAGTACCAT-3′	66	Passed
		Rev: 5′-CGAGGTCATCTCTGCCAAACTTG-3′	67	
		Probe: CAL Fluor Gold 540—BHQ1	-	
		5′-ACTGAGTCTAATGTTTAGGCCTTCCAGTGT-3′	70	
	CAT/ NM_009804	For: 5′-ACAACGCTGAGAAGCCTAAGA-3′	67	Passed
		Rev: 5′-GCTGAGCACCGGAGTTACAG-3′	67	
		Probe: CAL Fluor Red 610—BHQ2	-	
		5′-ACCTACACGCAGGCCGGCTCTC-3′	70	
	GAPDH/ NM_008084	For: 5′- GTGTCCGTCGTGGATCTGA-3′	67	Passed
		Rev: 5′-CACCACCTTCTTGATGTCATCATAC-3′	67	
		Probe: Quasar 670—BHQ2	-	
		5′-CGTGCCGCCTGGAGAAACCT-3′	70	
	ACTB/ NM_007393	For: 5′-GACAGGATGCAGAAGGAGATTACTG-3′	66	Passed
		Rev: 5′-GCTCAGGAGGAGCAATGATC-3′	66	
		Probe: Quasar 705—BHQ2	-	
		5′-TGGCTCCTAGCACCATGAAGATCA-3′	70	
#2	*DDB2*/ NM_028119	For: 5′-GGCCACAGCCTCCATAGATC-3′	66	Passed
		Rev: 5′-GTGAGGCAGTGAGTAGAGGAA-3′	67	
		Probe: FAM—BHQ1	-	
		5′-AGTGAAGATTTGGGACCTGCGCCAAA-3′	70	
	BBC3/ NM_010786	For: 5′-TGCGTTAAAGCTTCAGAGACAAG-3′	66	Passed
		Rev: 5′-CCCACCGCCTCCTATTCAAA-3′	67	
		Probe: CAL Fluor Gold 540—BHQ1		
		5′-CAAGAACACTCCTCCCACGTGACA-3′	70	
	PPARG/ NM_011146	For: 5′-CTCAGGCAGATCGTCACAGA-3′	66	Failed, excluded
		Rev: 5′-TCAGCGGGTGGGACTTTC-3′	66	
		Probe: CAL Fluor Red 610—BHQ2	-	
		5′-TGCAGCTACTGCATGTGATCAAGAA-3′	69	
	GAPDH/ NM_008084	For: 5′-CCTGGAGAAACCTGCCAAGTATG-3′	67	Passed
		Rev: 5′-AGCCCAAGATGCCCTTCAG-3′	67	
		Probe: Quasar 670—BHQ2	-	
		5′-CAAGAAGGTGGTGAAGCAGGCATCT-3′	70	
	ACTB/ NM_007393	For: 5′-GTGACGTTGACATCCGTAAAGAC-3′	66	Passed
		Rev: 5′-GCCTGGGTACATGGTGGTA-3′	66	
		Probe: Quasar 705—BHQ2	-	
		5′-ATGCCAACACAGTGCTGTCTGGT-3′	70	
#3	*DDIT3*/ NM_007837	For: 5′-GAGCTGGAAGCCTGGTATGAG-3′	66	Passed
		Rev: 5′-GGACGCAGGGTCAAGAGTAG-3′	66	
		Probe: FAM—BHQ1	-	
		5′-TGCAGGAGGTCCTGTCCTCAGAT-3′	70	
	MDM2/ NM_010786	For: 5′-GTGGGCTGCAGAGAAGAC-3′	66	Passed
		Rev: 5′-CATGCGTGCATCCATTTGAAGATC-3′	66	
		Probe: CAL Fluor Gold 540—BHQ1	-	
		5′-CAGCCAGTAAAGGCACCTGCTGT-3′	70	

Table S1. *Cont.*

5plex ID	Gene Name/ID [a]	Sequence/Probe Type	T_m (°C)	Validation Result
#3	*TNFSF10*/ NM_009425	For: 5′-AGGCAACTGGAGCTAAAGACAAG-3′	66	Failed, excluded
		Rev: 5′-CCACCCTGTTCCTCACAGATTC-3′	67	
		Probe: CAL Fluor Red 610—BHQ2	-	
		5′-TAAGCTGGTGTACCCCGATGGAGC-3′	70	
	GAPDH/ NM_008084	For: 5′-CCTGGAGAAACCTGCCAAGTATG-3′	67	Passed
		Rev: 5′-AGCCCAAGATGCCCTTCAG-3′	67	
		Probe: Quasar 670-BHQ2	-	
		5′-CAAGAAGGTGGTGAAGCAGGCATCT-3′	70	
	ACTB/ NM_007393	For: 5′-GTGACGTTGACATCCGTAAAGAC-3′	66	Passed
		Rev: 5′-GCTCAGGAGGAGCAATGATC-3′	66	
		Probe: Quasar 705—BHQ2	-	
		5′-ATGCCAACACAGTGCTGTCTGGT-3′	70	
#4	*FOXO3A*/ NM_019740	For: 5′-GCAAAGCAGACCCTCAAACTG-3′	66	Passed
		Rev: 5′-CGGCGTGGGAGTCTCAAAG-3′	66	
		Probe: Quasar 705—BHQ2	-	
		5′-AAGACCTACAGAGAAAACCCTTTGCCAA-3′	69	
	GADD45α/ NM_007836	For: 5′-GACGAACCCACATTCATCACA-3′	66	Passed
		Rev: 5′-GGGCACCCACTGATCCAT-3′	66	
		Probe: CAL Fluor Gold 540—BHQ1	-	
		5′-TGGAAGGATCCTGCCTTAAGTCAACTTA-3′	68	
	DDB1/ NM_015735	For: 5′-CACACTGAGCGAAAGACAGAAC-3′	66	Passed
		Rev: 5′-GGCGGCTGATATCTAGGAAAC-3′	65	
		Probe: CAL Fluor Red 610—BHQ2	-	
		5′-CAGCCACAGGCTTCATCGATGGT-3′	70	
	GAPDH/ NM_008084	For: 5′-GCCAAGGTCATCCATGACAAC-3′	67	Passed
		Rev: 5′-TCACGCCACAGCTTTCCAG-3′	67	
		Probe: Quasar 670—BHQ2	-	
		5′-TGTGGAAGGGCTCATGACCACA-3′	70	
	ACTB/ NM_007393	For: 5′-GACAGGATGCAGAAGGAGATTAC-3′	66	Passed
		Rev: 5′-GCTCAGGAGGAGCAATGATC-3′	66	
		Probe: Quasar 705—BHQ2	-	
		5′-TGCTCTGGCTCCTAGCACCA-3′	69	
#5	*HSPA1B*/ NM_010478	For: 5′-CCATCGAGGAGGTGGATTAGAG-3′	65	Passed
		Rev: 5′-GTGCCCAAGCAGCTATCAAG-3′	66	
		Probe: Quasar 705—BHQ2	-	
		5′-CTGCTGGCTCTCCCGGTGTG-3′	70	
	G6PC/ NM_008061	For: 5′-GCAAGCCCAGGCTAGAGATC-3′	67	Failed, excluded
		Rev: 5′-TTTCCCAGCCGGAAGATTC-3′	66	
		Probe: CAL Fluor Gold 540—BHQ1	-	
		5′-TGAGAATGCTCTTGCGGTGCA-3′	70	
	BCL2L11/ NM_207681	For: 5′-TTGCCAAGGCCACTTGTG-3′	66	Passed
		Rev: 5′-GGGAGCCGAATGGTATAGGTAAG-3′	66	
		Probe: CAL Fluor Red 610—BHQ2	-	
		5′-CAGGACACTCGCTGGCTACCG-3′	70	
	GAPDH/ NM_008084	For: 5′-CCTGGAGAAACCTGCCAAGTATG-3′	66	Passed
		Rev: 5′-GCCCAAGATGCCCTTCAGT-3′	67	
		Probe: Quasar 670—BHQ2	-	
		5′-CAAGAAGGTGGTGAAGCAGGCATCT-3′	70	
	ACTB/ NM_007393	For: 5′-GTGACGTTGACATCCGTAAAGAC-3′	66	Passed
		Rev: 5′-GCTCAGGAGGAGCAATGATC-3′	66	
		Probe: Quasar 705—BHQ2	-	
		5′-ATGCCAACACAGTGCTGTCTGGTG-3′	70	

[a] NCBI RefSeq accession number. # designates the number of an assay. Abbreviations: BHQ, black hole quencher; FAM, 5-carboxyfluorescein.

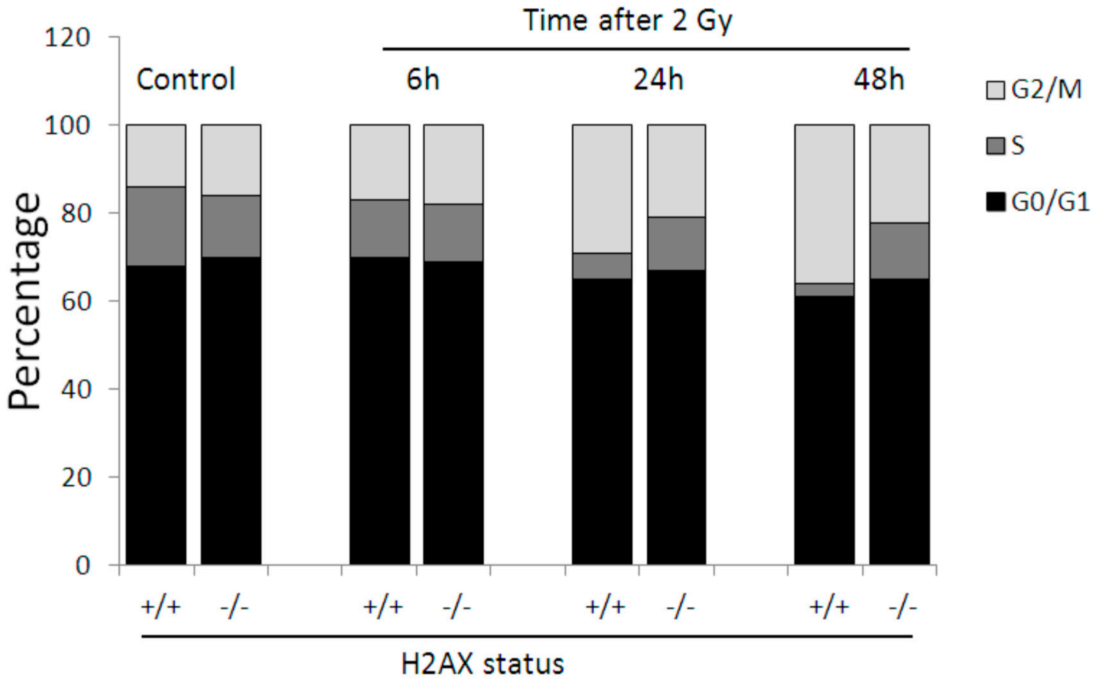

Figure S1. Distribution of cell cycle phases in MEF *H2AX*$^{+/+}$ *vs.* MEF *H2AX*$^{-/-}$ cells under control and irradiated condition. Subconfluent cells were sham- or γ-irradiated with 2 Gy followed by fixation in 70% methanol at indicated time points and stored at −20 °C. Fixed cells were subsequently rehydrated, stained with DAPI (4′,6-diamidino-2-phenylindole) and analyzed using the Cell Lab Quanta SC flow cytometer (Beckman Coulter). DAPI (DNA content) fluorescence were triggered by an mercury arc lamp and detected and measured in the FL1 (525BP) channel. Readings from at least 5000 cells were collected and analyzed by Quanta Analysis software (Beckman Coulter) to calculate fraction of cells in various cell cycle phases. Data are the means of two independent experiments.
